# European sovereign debt control through reinforcement learning

**DOI:** 10.3389/frai.2025.1569395

**Published:** 2025-06-18

**Authors:** Tato Khundadze, Willi Semmler

**Affiliations:** ^1^Department of Economics, The New School for Social Research, New York, NY, United States; ^2^Business Administration and Economics, Bielefeld University, Bielefeld, Germany; ^3^Economic Frontiers Program, IIASA, Laxenburg, Austria

**Keywords:** fiscal policy, deep reinforcement learning, Euro area, NMPC, machine learning, Soft Actor-Critic, actor critic algorithm

## Abstract

The resilience of economic systems depends mainly on coordination among key stakeholders during macroeconomic or external shocks, while a lack of coordination can lead to financial and economic crises. The paper builds on the experience of global and regional shocks, such as the Eurozone crises of 2009–2012 and the economic disruption resulting from COVID-19, starting in 2020. The paper demonstrates the importance of cooperation in monetary and fiscal policies during emergencies to address macroeconomic non-resilience, particularly focusing on public debt management. The Euro area is chosen as the sample for testing the models presented in the paper, given that its resilience is heavily dependent on cooperation among different actors within the region. The shocks affecting nations within the European Union are asymmetric, and the responses to these shocks require coordination, considering heterogeneous economic structures, levels of economic development, and policies. We develop a macroeconomic modeling framework to simulate fiscal and monetary policy interactions under a cooperative regime. The approach builds on earlier nonlinear control models and incorporates modern reinforcement learning techniques. Specifically, we implement the Soft Actor-Critic algorithm to optimize policy responses across key variables including inflation, interest rates, output gaps, public debt, and government net lending. We demonstrate that the Soft Actor-Critic algorithm provides comparable or, in some cases, better solutions to multi-objective macroeconomic optimization problems, in comparison to Nonlinear Model Predictive Control (NMPC) algorithm.

## 1 Introduction

The Euro area economy can be viewed as a large, complex system whose resilience depends on a combination of external and internal factors. For modeling purposes, it is valuable to identify and isolate the key elements that contribute to the system's stability and to examine how these elements evolve within a simulated environment. Modern macroeconomic literature and existing policy studies have provided four key directions that are important from the standpoint of macroeconomic resilience in such a complex economic system as the European Union. As key macroeconomic challenges are seen: (i) the decline in competitiveness and slow pace of growth, (ii) long-term debt sustainability, (iii) the need for a green transition, and all this (iv) in the context of an uneven economic development within the EU (Fagerberg et al., 2016).

The key question is how to achieve the long-term goals of sustaining high growth rates while keeping debt levels manageable. According to the Maastricht Treaty, a fiscal rule in the European Union stipulates that government debt should not exceed 60% of GDP, and government deficits should not surpass 3% of GDP. If a country's debt-to-GDP ratio breaches this rule, the recently postulated adjustment rule is that the respective country is expected to gradually reduce the ratio until it reaches those thresholds. However, those fixed and adjustment rules are schematic and represent a highly debated general framework, that is challenged in recent policy debates (Grauwe, 2025). In fact there could be multiple regimes in terms of understanding debt sustainability. A while ago De Grauwe provided the explanation of the emergence multiple equilibria in the context of the European Union. According to him, considering the monetary union, the member countries can't issue debt in their own currency, which means that they are in the same position as many developing countries where local financial markets are not sufficiently developed, so that governments can borrow money in a local currency (De Grauwe, 2011).

Furthermore, Blanchard (2022) applies empirically such a multiple equilibria framework. In his view there are “good” and “bad” equilibria, in terms how they may be able to become resilient impacting an economy. The good equilibria are sustainable and self-stabilizing the debt level. This occurs under high economic growth rates, which help maintain low risk premia and lower effective interest rates. In the good equilibria, debt ratios tend to converge toward sustainable steady state levels, which implies that this level of debt does not pose the risk of financial stability (Blanchard, 2022). On the other hand bad equilibria are characterized by unsustainable debt dynamics—high levels of debt lead to destabilization of the system. As Semmler and Young (2024) explain, when the macroeconomic system is in a bad equilibrium, macroeconomic non-linearities are playing greater role, specifically, thresholds and tipping points, that can be source of sudden disruptions in the macro economy. The self-reinforcing loop works in the following way: higher risk premia and interest rates exacerbate the debt situation and the high debt requires higher risk premia.

In light of a perceived “secular stagnation,” EU countries are facing persistently slow growth, characterized by relatively high savings rates but slowed down private and public investment rates. Blanchard (2022) suggests that this is in part due to a preference for safe assets. This imbalance between savings and investment has pushed down the neutral interest rate—the hypothetical rate that keeps the economy at a knife edge problem of neither too high nor too low inflation and growth rates. Given private sector expectations for returns are diminishing, actual interest rates tend to fall and central banks, whose objectives are to maintain stable prices and reasonable growth, often respond by cutting rates further. However, given the long delay effects of interest rate changes even with lower rates, private consumption and investment demands may remain sluggish.^1^ Another factor possibly contributing to low interest rates is the growing demand for safe assets, such as government bonds. This trend drives up the price of these assets, putting downward pressure on their yields (interest rates).

The neutral interest rate can then fall below the the growth rate, which means that the cost of servicing debt has decreased. As a result, monetary policy may face the zero bound of the interest rate and has become less effective in managing the economy, and fiscal policy is likely to take on a more prominent role. Lower neutral rates also mean lower debt servicing costs, which creates more space for public borrowing. This traditional view of fiscal policy assumes that monetary policy can effectively manage the economy until the zero bound of the interest rate is reached and then fiscal policy can keep output close to its potential. On the other hand when private demand is strong, and growth rate and employment reasonable high, fiscal policy should focus on stabilizing debt (Blanchard, 2022).

On the other hand, if monetary policy is ineffective in closing the output gap, and fiscal policy faces unsustainable debt often budget consolidation drive fiscal policy with often adverse effects on output and employment. Budget consolidations might be achieved but at considerable cost.^2^ Fiscal policy can push the neutral rate above the upper bound, but still keep the actual interest rate below the growth rate and therefore enabling to pursue debt sustainability. On the other side, in periods of low interest rates and weak private demand, governments may be forced to run deficits to keep output close to its potential, which in turn can increases the debt-to-GDP ratio because also high risk premia can arise driving up the borrowing rate. These are mechanisms we want to capture in our macrodynamic model with good and bad debt equilibria.

The another key challenge not to be neglected in this context is the problem related with slow productivity growth within European Union. In comparison with the US the growth rate of the EU was lower, while also China had much higher growth, rising world export shares, and in the process of becoming an industrial powerhouse. As recently published report by Draghi (2024) suggests, the key factors which explain the gap in GDP growth rate of the US—the EU being deficient in public investments and productivity growth: specifically, the report suggests that 70 percent of in GDP per capita gap between the EU and USA can be explained by relatively low rate of productivity in the EU. The EU is losing its competitiveness on the global market in particular in sectors relevant for the green transition, for example digitization, AI innovative investments, electrical vehicles, batteries and so on. Much became more prevalent after the COVID-19 pandemics. The challenge comes from the US and also Chinese companies, making the foreign demand for EU products decreasing (Draghi, 2024).

The Draghi report highlights these factors: the EU is falling behind in technological development. The US has seven super high-tech companies with asset values exceeding a trillion dollars, while the EU has none. Only 4 companies among global tech companies are from the EU. The Draghi report highlights if the current EU labor productivity growth rate stays the same (on average 0.7% since 2015), it would be enough to keep the GDP constant only until 2050 (Draghi, 2024). While there is more optimism in terms of interest rates, some express concerns about the slower growth rates. Under the slower growth rates, the EU debt levels may become untenable and the EU maybe forced to slow down its plans in terms decarbonization or other goals (Draghi, 2024). As Blanchard (2022) predicts that future growth rates above the interest rate are crucial for debt sustainability and for sovereign debt control.

With this paper, we contribute to the literature on simulating debt dynamics and debt sustainability in the Eurozone countries, and also contribute to the literature on the application of deep reinforcement learning macroeconomics. It is important to utilize modern economic models, including simulations powered by machine learning, to address macroeconomic management challenges. European sovereign debt control can get the help of machine learning. When interest rates are lower than the growth rate, governments can manage higher debt levels without encountering fiscal difficulties, as economic growth helps offset the debt burden. This allows also for running primary deficits while maintaining stable debt levels. However, this condition is not guaranteed in the long term: Unexpected shocks could raise interest rates above the growth rate, leading to move closer to unsustainable debt levels and a “bad” debt equilibrium. The paper using Deep Reinforcement Learning and Non-linear Model Predictive Control (NMPC) provides a novel approach to solving a multi-objective macroeconomic problem aimed at minimizing the deviation of multiple macroeconomic state variables from their target levels.

Deep Reinforcement Learning (DRL) allows to introduce a new macroeconomic framework and study macrodynamic problems alongside an established NMPC algorithm which was known for a while in economic literature. While based on different principles, the NMPC method and Deep Reinforcement Learning (e.g., the Soft Actor-Critic algorithm) can address similar problems in dynamic economic systems—one operating within a deterministic framework, the other within a stochastic one. Both have as multi-period target to minimize deviations in the inflation rate, output gap, and debt levels from their respective targets under a cooperative scenario. In this scenario, monetary and fiscal policies are synchronized between two groups (North and South) of EU countries. Simulations of this cooperative scenario, comparing NMPC and the stochastically oriented Deep Reinforcement Learning, offer insightful perspectives on how key macroeconomic variables may evolve through objective function optimization and policy learning, aiding in sustainable sovereign debt control.

The second section of the paper summarizes the stylized facts regarding debt sustainability and other trends of the EU over the last 30 years. Specifically, the variables include the interest rate for the European Central Bank's main refinancing operations (MRO), government net lending, consolidated gross government debt, the Harmonized Index of Consumer Prices (HICP), and the output gap. The third section provides a macromodel based on the idea of a cooperative macro-dynamic solution for EU countries. The fourth section offers a brief review of Non-linear Model Predictive Control and various forms of Deep Reinforcement Learning, which are used to solve the macro model with possibly good and bad debt equilibria as presented in the previous section. The final section presents the simulation results for NMPC and the Soft Actor-Critic (SAC) algorithm, demonstrating how the state variables may evolve under the cooperative scenario within EU countries and studies how the bad debt equilibrium can be avoided.

## 2 Stylized facts

For modeling purposes, it is valuable to identify and isolate the key elements that contribute to the system's stability and to examine how these elements evolve within a simulated environment. The variables considered for describing the macroeconomic system include the interest rate for the European Central Bank's main refinancing operations (MRO), government net lending, consolidated gross government debt, the Harmonized Index of Consumer Prices (HICP), and the output gap. Describing the dynamics of the given variables is essential for detecting stylized facts in an uncooperative scenario. In this context, “uncooperativeness” refers to the absence of a common or cooperative fiscal policy among the North and South country groups defined below.

Following the approach of Semmler and Haider (2018), we divide the Euro area into two regions: North and South, encompassing countries such as Germany, France, Spain, and Italy. The North-South aggregates are constructed based on their respective GDPs. We examine these variables from a historical perspective to identify and capture stylized facts. From the perspective resilience it is important to observe how the economic variables evolve during the shock periods.

**Figure 1:**
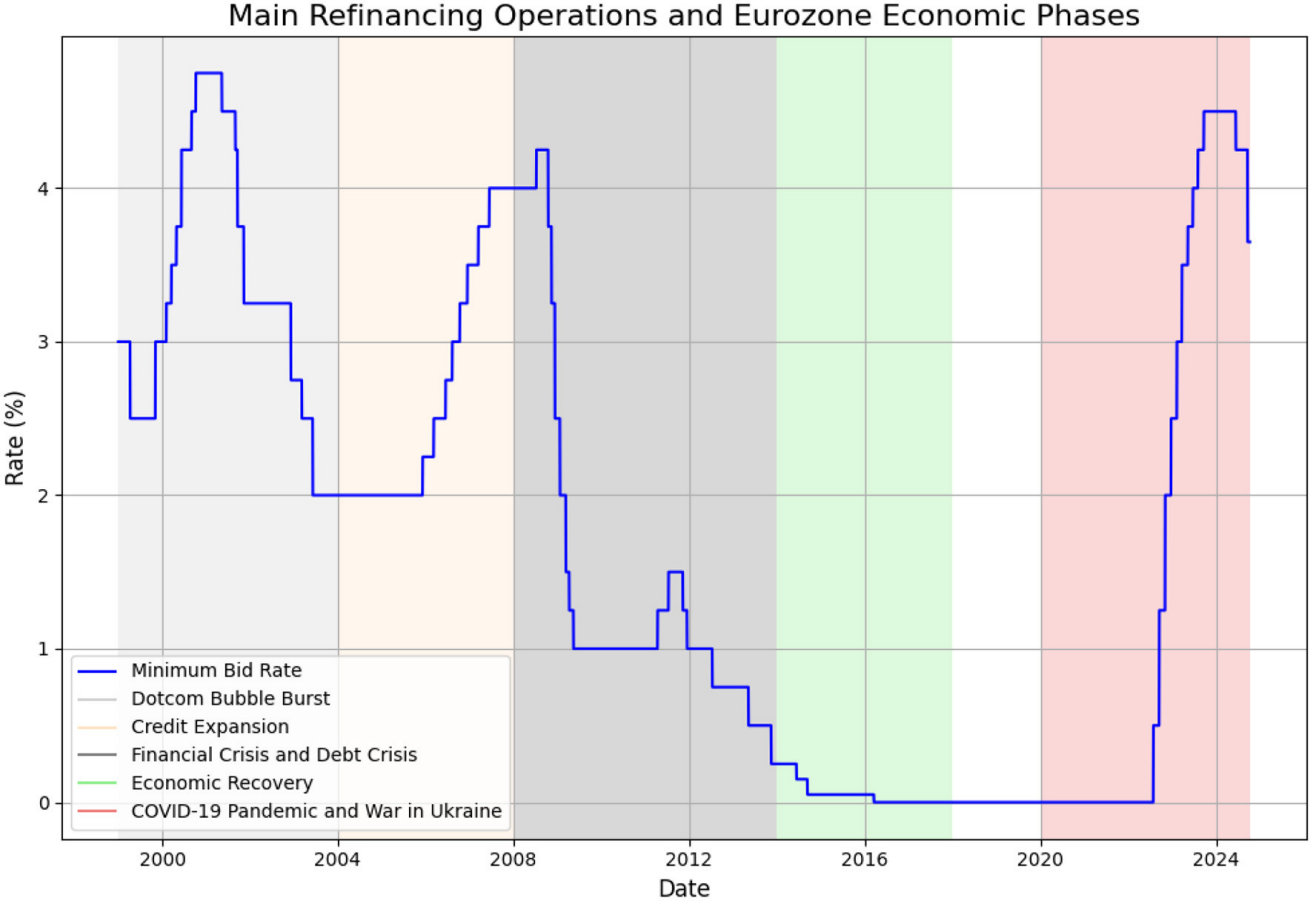
Main refinancing operations.

The Eurozone's recent macroeconomic history can be divided into several episodes. For instance, Hartmann and Smets (2018) provide four phases from 1999 to 2017. Specifically, the phases in Figure 1 include the following: from 1999 to 2003, involved a slowdown in growth after the dotcom bubble burst, accompanied by a weak euro. The second phase, from 2004 to 2007, was marked by a surge in money and credit expansion, stable inflation, and accelerating economic growth. This increase corresponds with a period of robust economic growth and inflationary pressures in the Eurozone, resulting in relatively high rates until 2008.The third phase, from 2008 to 2013, saw a double-dip recession, caused by the U.S. financial crisis and the euro area sovereign debt crisis. The final phase, from 2014 to 2017, was characterized by economic recovery in a low-inflation environment. A substantial decline in the MRO rate begins in 2008, coinciding with the global financial crisis and the subsequent Eurozone sovereign debt crisis.

In response to the economic downturn, the ECB significantly reduced its rates, lowering them to approximately 1.00% by 2009. After a brief increase in 2011, the rate was further reduced to 0.75% by 2012 as the crisis intensified, particularly in countries like Greece, Spain, and Italy. From 2013 to 2019, the graph depicts a period of sustained low interest rates, eventually reaching 0.00% by 2016. This period reflects the ECB's approach to addressing low inflation and economic stagnation in the Eurozone, employing a combination of low rates and unconventional monetary policies, such as quantitative easing (Hartmann and Smets, 2018). Hartmann and Smets classification of ECB policy can be further extended in order to cover episodes COVID-19 pandemic and war in Ukraine. During the COVID-19 pandemic in 2020 and 2021, the rate remained at 0.00%, demonstrating the ECB's commitment to maintaining liquidity and supporting the economy amid severe economic contractions. The ECB utilized combination of conventional and unconventional monetary policy mix during the pandemic. This policy mix included Pandemic Emergency Purchase Programme (PEPP), under which the ECB purchased 750 billion worth of securities (De Guindos and Schnabel, 2022). In parallel, the ECB continued and improved other non-conventional measures, such as targeted long-term refinancing operations (TLTROs). Specifically, the objective of Targeted Long-Term Refinancing Operations was to provide sufficient liquidity to the real sector and also to improve lending conditions (Fernndez et al., 2021).

A significant upward trend in interest rates begins in mid-2022, reflecting a series of rapid rate hikes by the ECB in response to rising inflation. By September 2023, the MRO rate reached 4.50%, representing one of the most rapid increases in the ECB's history. This adjustment reflects the ECB's efforts to counter inflationary pressures caused by the war in Ukraine, supply chain disruptions, and the energy crisis (Maurya et al., 2023). A slight reduction to 4.25% in 2024 indicates a cautious adjustment as inflationary concerns start to moderate but remain a key focus.

Overall, the Figure 1 captures the ECB's evolving monetary policy strategies, from pre-crisis moderate rate hikes, sharp cuts during the Eurozone crisis, a prolonged period of near-zero rates amid economic stagnation and the COVID-19 pandemic, to aggressive rate increases in response to post-pandemic inflation, geopolitical tensions and fossil fuel prices rising.

**Figure 2:**
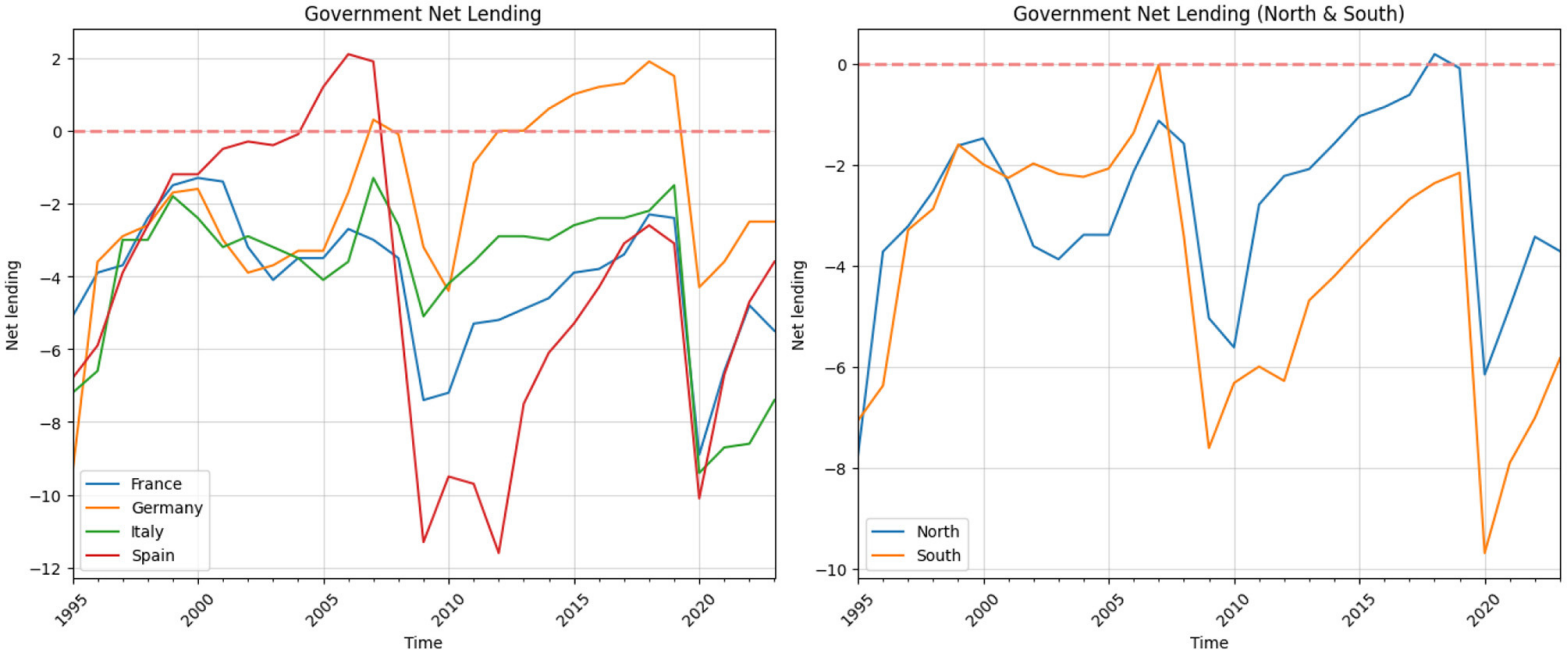
Government net lending.

There are two important thresholds regulated by the EU Stability and Growth Pact (SGP): Government Deficit Limit and Government Debt Limit.^3^ The Figure 2 of government net lending in France, Germany, Italy, and Spain between 1995 and 2023 shows different paths in their financial situations, influenced by national policies and broader economic events like the 2008 global financial crisis and the COVID-19 pandemic. One important aspect which can be regarded as stylized fact from this picture is that, the EU countries breach the deficit limit considerably, when there are strong economic downturns. In the given period, two such significant economic downturns appeared, such as Global Financial Crises in 2008 and the economic crises which follow COVID-19 pandemics in 2019. The second stylized fact is related to the scale of the deficit rule.

In France, the government deficit started at around 5.1% of GDP in 1995. Over the next decade, the government worked to improve its financial situation, bringing the deficit closer to balance by the early 2000s. However, there were some setbacks, such as in 2003, when the deficit increased to about 4.1%. Despite these ups and downs, France managed to keep its financial position relatively stable until the 2008 financial crisis. This crisis caused a large increase in the deficit to 7.4% in 2009, mainly because of lower revenues during the recession and higher government spending to support the economy. After this, France slowly started to recover, reducing the deficit to 2.4% by 2019. However, the COVID-19 pandemic caused new problems, increasing the deficit to 8.9% in 2020. By 2023, the deficit had improved a bit to 5.5%, but it was still relatively high.

Germany began with a large deficit of 9.4% of GDP in 1995 but saw steady improvement over the next decade. By 2007, Germany achieved a small surplus of 0.3%, thanks to strict control over spending and good economic conditions. The financial crisis in 2008 briefly pushed Germany back into a deficit of 3.2% in 2009, but the country quickly returned to a surplus from 2012 onward, reaching its highest point at 1.9% in 2018. The COVID-19 pandemic in 2020 caused another deficit of 4.3%, but Germany reduced this to 2.5% by 2023, showing effective management of its finances during the recovery.

Italy faced many financial problems throughout the period. Starting with a deficit of 7.2% in 1995, it managed to lower the deficit to 1.3% by 2007. However, Italy often struggled with its public budget, and the deficit remained above 3% of GDP. The financial crisis in 2008 made things worse, with the deficit reaching 5.1% in 2009. Italy's recovery was slow, and by 2019, the deficit was 1.5%. The COVID-19 pandemic in 2020 made the situation even worse, pushing the deficit to 9.4%. By 2023, the deficit was still high at 7.4%, showing that Italy continues to face deep economic challenges. Spain improved its financial situation significantly from 1995 to 2006, moving from a deficit of 6.8% to a surplus of 2.1% due to strong economic growth and reforms. However, the 2008 financial crisis caused the deficit to grow sharply to 11.3% in 2009, and it reached 11.6% again in 2012 during the Eurozone crisis. From 2014 to 2019, Spain worked hard to reduce its deficit, bringing it down to 3.1% by 2019. The COVID-19 pandemic in 2020 led to another large increase in the deficit to 10.1%, but Spain managed to lower it to 3.6% by 2023, although it was still higher than before the pandemic.

It is important to note that the EUs fiscal deficit rule has not been consistently adhered to before and after 2008, as illustrated in Figure 2. With the exception of Germany, most countries have frequently violated the rule. Second, fiscal positions appear to be subject to different regimes. Economic and exogenous shocks significantly affect the fiscal position of these nations. As shown in Figure 2, there are at least two distinct episodes—the 2008 Financial Crisis and the 2019 COVID-19 crisis—during which fiscal deficits increased sharply. However, it is evident that fiscal deficits respond differently to various shocks. For instance, although Germanys fiscal deficit rose in both episodes, the increase was more modest compared to other countries in the group. The fiscal policy response to shocks is largely influenced by automatic stabilizers: when output falls, lower tax revenues and increased transfers stimulate aggregate demand. The variation in fiscal responses can be attributed to several factors, including the nature of the shock and the specific vulnerabilities of each country. In general, the strength of automatic stabilizers depends on the degree of tax progressivity and the size of the transfer system. According to an OECD study, countries with more progressive tax systems and more generous transfer programs tend to have stronger automatic stabilizers that respond more aggressively to output declines (Maravalle and Rawdanowicz, 2020).

**Figure 3:**
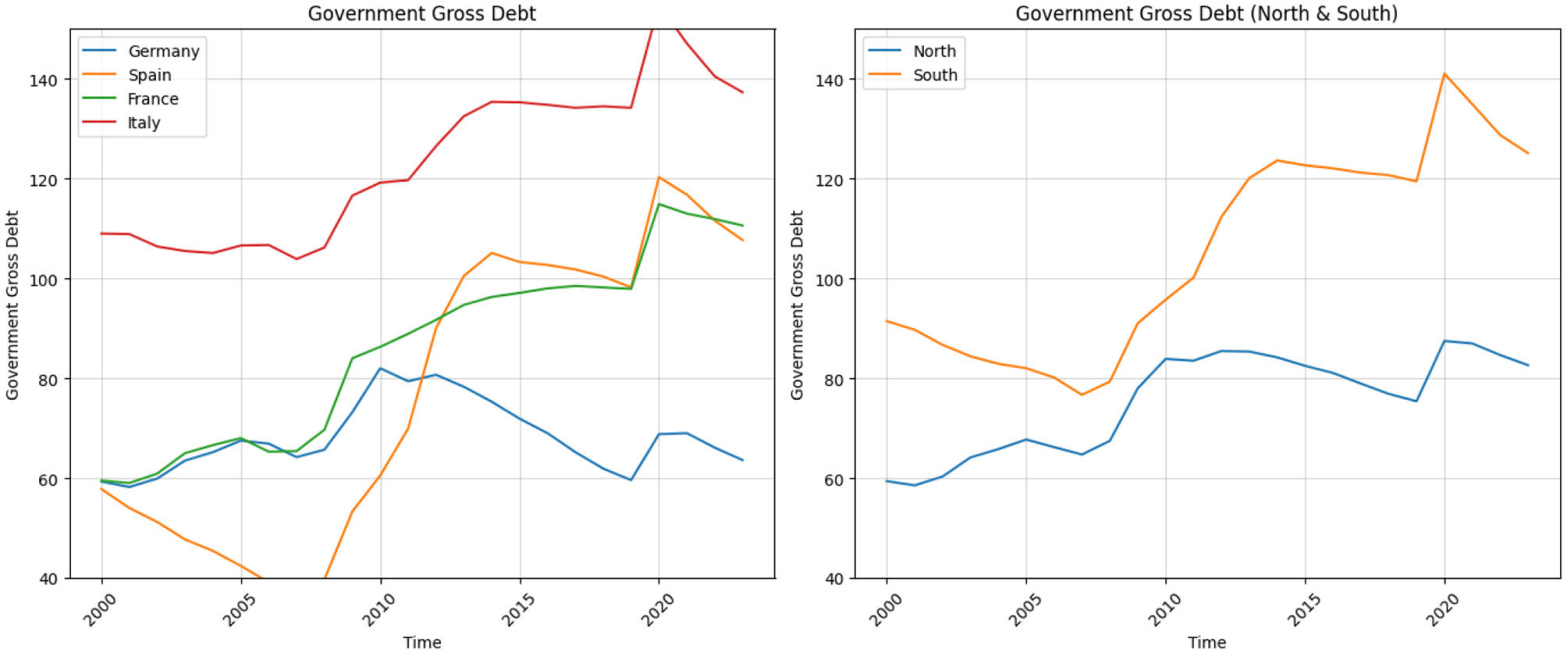
Government gross debt.

Another SGP ceiling is Debt-to-GDP ratio, which should not exceed 60%. Figure 3 illustrates the evolution of this indicator over the period of 2000–2023. The evolution of government gross debt as a percentage of GDP for Germany, Spain, France, and Italy from 2000 to 2023 illustrates distinct trends, particularly during key economic shocks. One important aspect, which is visible from this evolution is the trended behavior of this indicator since the Global Financial Crises for all countries described on the graph except Germany. The debt-to-GDP ratio decreases and plateaus up to 2019, however, there is a sudden spike after the COVID-19 pandemic.

For Germany, there is a steady rise in debt, reaching about 73% in 2009 due to the global financial crisis. The ratio peaks at 82% in 2010, reflecting increased borrowing and economic support measures, then gradually decreases. A noticeable upward jump appears in 2020, pushing the debt ratio to nearly 69% during the COVID-19 pandemic, before decreasing again to around 64% by 2023. France's debt ratio steadily increases from the early 2000s, with a marked rise starting in 2008, reaching around 84% by 2009 during the financial crisis. The ratio continues to climb, peaking at ~115% in 2020 due to the pandemic, with a slight reduction to about 111% by 2023.

Spain shows a steep rise from around 36% in 2007 to over 100% by 2014, driven by the financial and Eurozone debt crises. After some stabilization, another sharp increase is visible in 2020, where the debt ratio jumps to 120% due to pandemic-related spending, followed by a gradual decline to ~108% by 2023. Italy starts high at 109% in 2000 and remains elevated, with a significant increase during the global financial crisis, reaching around 119% in 2010. Another sharp rise occurs during the Eurozone debt crisis, peaking at 135% by 2014. In 2020, the debt ratio surges to 155% due to the COVID-19 pandemic, followed by a gradual decline to 137% by 2023.

It is important to note that after 2012, as shown in Figure 3, the debt-to-GDP ratio began to stabilize in both the Northern and Southern regions of the Eurozone—a trend that persisted until the onset of the COVID-19 crisis. In this context, the interest rate on government debt (safe real interest rate), not only reflects average rates on short- and long-term government bonds, but also includes risk premium. After the financial crises in 2009–2010 period, this effective interest rate start to increase relative to the economy's growth rate. The resulting gap between interest rates and growth created challenges for managing rising debt levels. In 2012, Mario Draghi intervened decisively to “rescue the euro" with his now-famous “whatever it takes" speech (Draghi, 2012). He initiated the purchase of Greek and Italian treasury bonds, which helped restore investor confidence, stabilize bond prices, and prevent capital flight. As a result, the risk premia embedded in sovereign yields decreased significantly.

**Figure 4:**
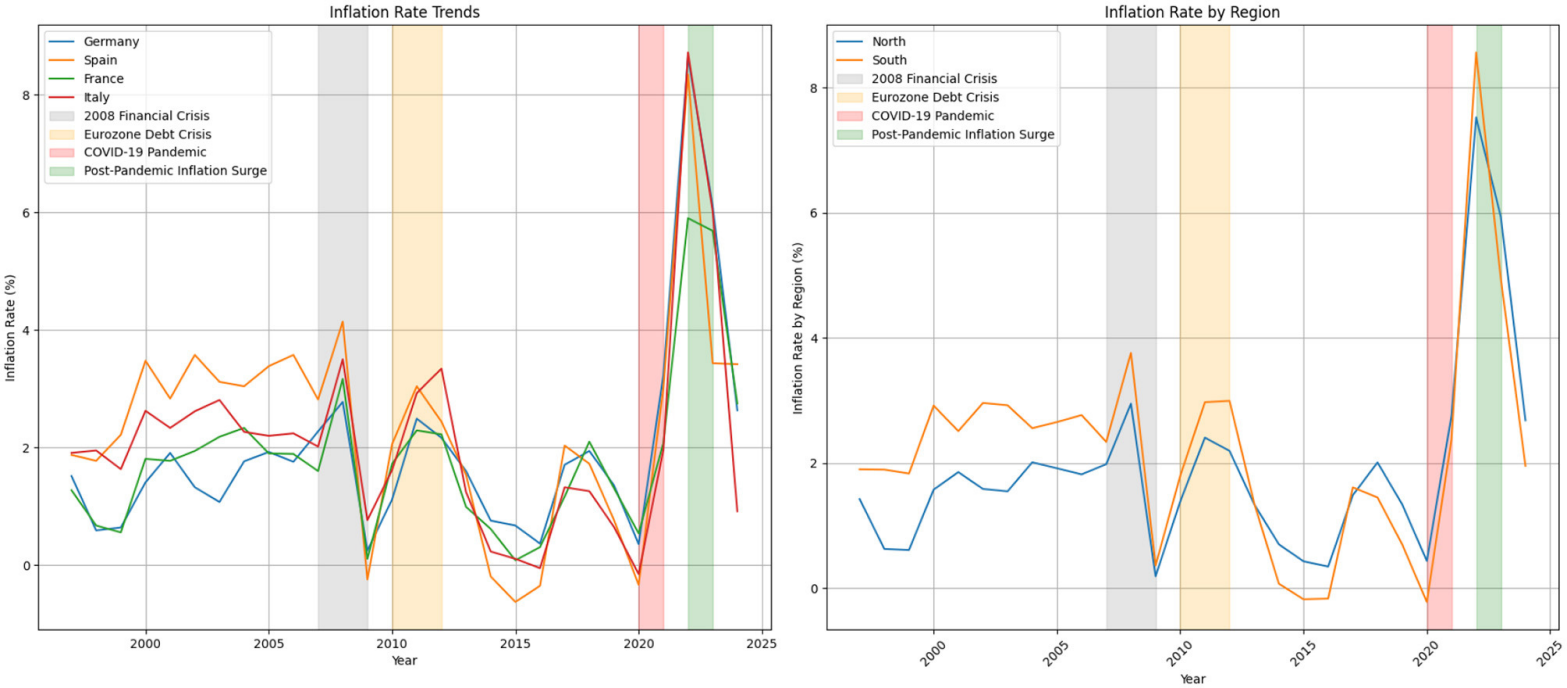
HICP annual change.

In terms of the inflation rate, there are three general trends that emphasize three key periods: a rise during the global financial crisis around 2008–2009, another increase during the Eurozone debt crisis in the early 2010s, and a pronounced spike after 2020 due to the COVID-19 pandemic and bottle necks, with varying levels of recovery afterward. Figure 4 illustrates inflation trends in Germany, Spain, France, and Italy from 1997 to 2024, reflecting the impacts of various economic shocks and policy responses over time.

During the decade preceding the 2008 financial crisis, inflation rates were relatively stable across these countries. Spain experienced slightly higher inflation, averaging around 3% in the early 2000s, partly due to rapid economic growth and a housing boom. In contrast, Germany maintained a lower inflation rate, averaging ~ 1.5% during the same period, reflecting its conservative monetary policies and economic stability. France and Italy showed moderate inflation trends, with France's rate averaging around 1.8% and Italy's rate decreasing gradually from about 2.6% in 2000 to 2.0% by 2007, as it aligned its fiscal policies with broader European economic standards.

The 2008 financial crisis marked a significant turning point. Inflation rates spiked in 2008, with Germany reaching 2.8%, Spain peaking at 4.1%, France at 3.2%, and Italy at 3.5%. This increase was driven by sharp changes in demand and uncertainty in financial markets. By 2009, inflation had fallen dramatically, with Germany at 0.3%, Spain at -0.2%, France at 0.1%, and Italy at 0.8%, reflecting the deep recession across Europe. In response, governments and the central bank implemented various fiscal stimulus and monetary easing measures, leading to diverse inflation trajectories in the following years.

Between 2010 and 2012, during the Eurozone debt crisis, inflation remained volatile. The crisis led to austerity measures, bailout packages, and increased financial market stress. Spain's inflation remained high, averaging around 3.0%, while Italy's inflation peaked at 3.3% in 2012. In contrast, Germany's inflation remained relatively controlled, staying around 2.2% due to its stronger fiscal position. France also maintained a moderate inflation rate, averaging ~ 2.2%.

In 2020, the COVID-19 pandemic first caused inflation rates to decline sharply, with Germany at 0.4%, Spain at -0.3%, France at 0.5%, and Italy at -0.2%. The economic disruptions caused by lock downs, reduced consumer spending, and supply chain issues led to these decreases. However, by 2021, inflation began to rise again due to pent-up demand and supply constraints, with Germany reaching 3.2%, Spain at 3.0%, France at 2.1%, and Italy at 2.0%.

The period from 2022 to 2023 witnessed a significant surge in inflation, with Germany's rate peaking at 8.6%, Spain at 8.3%, Italy at 8.7%, and France at 5.9%. This was driven by supply chain disruptions, energy price increases, and geopolitical tensions, including the conflict in Ukraine, which affected global energy and food supplies. In response, the European Central Bank implemented tighter monetary policies and national governments undertook fiscal adjustments to curb inflation.

By 2024, inflation rates appeared to stabilize, though at varying levels: Germany at 2.6%, Spain at 3.4%, France at 2.8%, and Italy at 0.9%. This stabilization is likely due to policy tightening, improved supply chain conditions, and adjustments in economic expectations following the shocks of the previous years. The data highlight the varying economic conditions and policy responses in these major European economies in response to both external and internal challenges.

**Figure 5:**
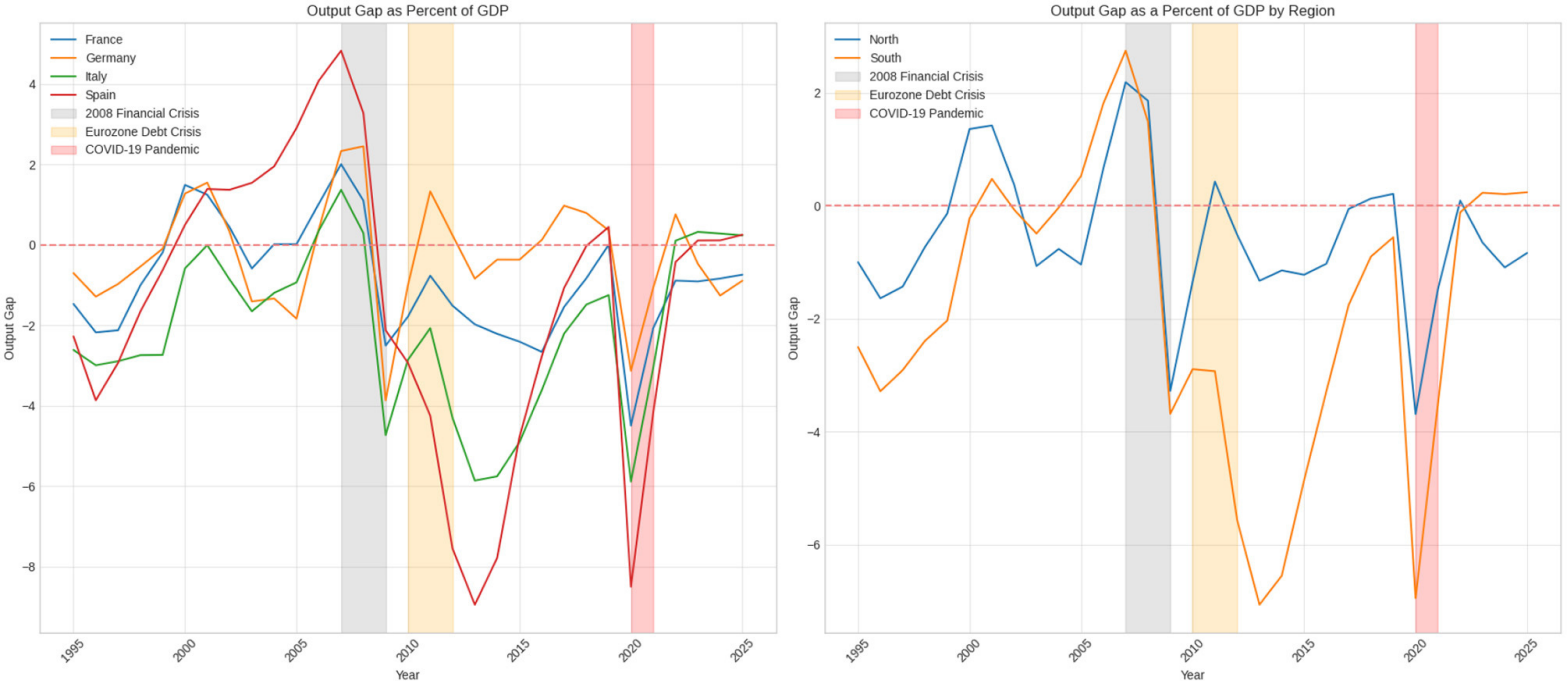
Output gap.

Figure 5 illustrates the output gap as a percentage of GDP for France, Germany, Italy, and Spain from 1995 to 2025. The output gap measures the difference between an economy's actual output and its potential output, helping to determine whether an economy is functioning above or below its full capacity. A positive output gap suggests that the economy is operating above its potential, while a negative output gap indicates that it is underperforming. Potential output is the maximum level of goods and services that a given economy can produce. It is a latent variable since it is not directly observable. The output gap reflects, in which phase of business cycle economy is located (Chen and Grnicka, 2020).

Between 1995 and the early 2000s, the output gaps for these four countries fluctuated around zero, which is consistent with periods of moderate growth and occasional economic slowdowns. During this period, Germany's output gap, after hovering near zero in the mid-1990s, shifted into positive territory around 2000, reaching a peak of 1.5% in 2001, indicating robust economic performance. Similarly, France experienced positive output gaps in the early 2000s, peaking at 1.5% in 2000. In contrast, Italy and Spain faced negative output gaps throughout the late 1990s, which highlights weaker economic conditions in these countries. On the other hand, between 2000 and 2008, Spain experienced rapid growth and, a positive output gap.

The 2008 financial crisis led to a sharp downturn. In 2009, the output gaps for all four countries fell significantly. Spain's output gap dropped to -2.1%, while Italy, France, and Germany saw their gaps decline to -4.7%, -2.5%, and -3.9%, respectively. This period reflects a severe contraction in economic activity across Europe, resulting in negative output gaps as actual production fell below potential output levels.

The recovery after the crisis was uneven among these countries. Germany quickly returned to a positive output gap, reaching 1.3% by 2011, supported by strong economic fundamentals and export growth. In contrast, France continued to show negative output gaps between 2010 and 2015, ranging from -0.8% to -2.4%. Italy and Spain also experienced persistent negative output gaps, particularly following the Eurozone debt crisis (2010–2012), which severely impacted their economies. In 2013, Spain's output gap fell to -9.0% and Italy's to -5.9%, reflecting deep recessions and the effects of austerity measures implemented during this period.

From 2016 to 2019, there were gradual improvements in all four countries. Both Germany and France succeeded in closing their output gaps, with Germany maintaining a positive output gap of ~ 1.0% in 2017, while France's output gap neared zero by 2019. Italy and Spain also saw their negative output gaps narrow, but they remained slightly below zero, indicating a slow recovery and ongoing structural economic challenges.

The COVID-19 pandemic in 2020 caused a further significant decline in the output gap across all four countries, as economic activity was heavily restricted due to lock downs and weakened demand. Spain's output gap decreased to -8.5%, while Italy, France, and Germany saw declines to -5.9%, -4.5%, and -3.1%, respectively. This period represents one of the most severe economic contractions since the financial crisis, with all countries struggling to regain their potential output levels.

With the gradual reopening of economies in 2021 and 2022, the output gaps began to show signs of recovery. By 2022, Germany's output gap was approaching zero, while France and Italy demonstrated gradual improvements. In 2023, Spain and Italy recorded slight positive output gaps of 0.1% and 0.3%, respectively, indicating modest economic recovery. However, Germany and France continued to experience negative output gaps, highlighting ongoing challenges in achieving pre-pandemic potential output levels.

## 3 Architecture of the cooperative policy and macroeconomic model description

The paper by Semmler and Haider (2018) provides a model-guided study of the above fluctuations using the formulation of the following objective function:


(1) $$\begin{array}{l} V(\pi ,y)=\min_{i_{t},f_{t}^{j}}\;\int_{0}^{T}e^{-\rho t}\{\lambda_{\pi }{\left(\pi_{t}-\pi_{s}\right)}^{2}+\lambda_{y}\\ \;\left[0.5{\left(y_{t}^{S}+y_{t}^{N}-y_{s}\right)}^{2}\right]+\\ \;\lambda_{l}\left[0.5{\left(d_{t}^{S}+d_{t}^{N}-d_{s}\right)}^{2}\right]\\ \;+\lambda_{i}{\left(i_{t}-i_{s}\right)}^{2}\}dt \end{array}$$

Equation 2 is the objective function, which has four quadratic penalty components: (1) deviation of the inflation rate π_*t*_ from the inflation target π_*s*_, (2) the average of the South and North output gaps ($y_{t}^{S}$ and $y_{t}^{N}$) from the EU-level output gap *y*_*s*_, (3) the deviation of the averaged public debt of South ($d_{t}^{S}$) and North ($d_{t}^{N}$) from the Euro area public debt target, and (4) element is the deviation of the interest rate from equilibrium interest rate, *i*_*s*_. The objective function is to be minimized with regards to interest rate and fiscal surplus. In other words, interest rate and fiscal surplus trajectory have to be chosen in a way that inflation rate, output, interest rate, and debt deviation from the targets are minimal. Given optimization problem contains a decision horizon T, over which the total cumulative cost should be minimized.

The dynamic constraints for the dynamic macroeconomic optimization problem are the following:


(2) $$\begin{array}{l} \overset{\cdot }{\pi }=\alpha_{1}\pi_{t}+\alpha_{2}\left(0.5\left(y_{t}^{S}+y_{t}^{N}\right)-y_{s}\right)-\pi_{s} \end{array}$$

(3) $$\begin{array}{l} {ẏ}^{S}=\beta_{1}y_{t}^{S}-\beta_{2}\left(i_{t}-\pi_{t}-r^{n}\right)-f_{t}^{S}-\beta_{3}{\left(f_{t}^{S}\right)}^{2} \end{array}$$

(4) $$\begin{array}{l} {ẏ}^{N}=\beta_{1}y_{t}^{N}-\beta_{2}\left(i_{t}-\pi_{t}-r^{n}\right)-f_{t}^{N}-\beta_{3}{\left(f_{t}^{N}\right)}^{2} \end{array}$$

(5) $$\begin{array}{l} {ḋ}^{S}=\gamma_{1}\left(d_{t}^{S}-d_{s}\right)-f_{t}^{S}+\epsilon_{t}^{S} \end{array}$$

(6) $$\begin{array}{l} {ḋ}^{N}=\gamma_{1}\left(d_{t}^{N}-d_{s}\right)-f_{t}^{N}+\epsilon_{t}^{N} \end{array}$$

Equation 2 describes the Phillips curve: It is the rate of change in prices in the euro area, which depends on the current inflation rate and the deviation of the output gap from the target. Equations 3, 4 describe how output gaps change over time, which is influenced by the present-level output gaps in both the South and North, as well as the discrepancy between the real interest rate and the natural interest rate, denoted as *r*^*n*^.

As for the Equations 5, 6, they describe the rate of change of the debt in South and North, which depend on the deviation of debt levels from public debt targets. The rate of change in debts also depends on the term of fiscal consolidation $f_{t}^{j}$, which according to Semmler and Haider (2018) reflects budget consolidations and fiscal deficits. Table 1 provides an overview of the parameter descriptions and their corresponding value ranges.

**Table 1:** Parameter descriptions and values.

**Parameter description**	**Parameter value**
Penalty on the deviation of inflation from the target π_*s*_	λ_π_
Penalty on the deviation of output from the euro-area output gap *y*_*s*_	λ_*y*_
Penalty on the deviation of public debt from the euro-area public debt target *d*_*s*_	λ_*l*_
Penalty on the deviation of the interest rate from the steady state interest rate *i*_*s*_	λ_*i*_
Reaction coefficient in the Phillips curve equation	α_1_>0, α_2_>0
Reaction coefficients in the output gap dynamics	β_1_, β_2_, β_3_>0
Mean-reversion parameter in the debt evolution equations	γ_1_ < 0;*or*>0 (depending on stable and unstable regimes, respectively)
Shock variables in the debt evolution equations	$\epsilon_{t}^{S},\epsilon_{t}^{N}$
Decision horizon for the optimization problem	*T*
Natural rate of interest	*r* ^*n*^

The Parameter which require more careful consideration in terms of public management is γ_1_, which is unique coefficient for the South and North debt equations. First, the value of γ_1_ coefficients should be < 0 (and absolute value should be < 1), in order to make sure the mean reverting character of the debt equations is obtained. Considering this defined range of the γ_1_ coefficient, debt equation will be stable even in the scenario, when the fiscal surplus equals to 0. Second within the framework of the differential Equations 5, 6, it is assumed that the γ_1_ coefficient is positive when *r*>*g*, with *r* the actual interest rate, resulting in debt instability in the sense of Blanchard (2019). This instability is often addressed through austerity measures, requiring $f_{t}^{S}$ and $f_{t}^{N}$ to take positive values, having a negative sign in front of them.

The difference between economic growth (g) and interest rates (specifically, long-term government bonds) (i) affects the public debt dynamics. According to Blanchard (2019), when *r*−*g* < 0, public debt may not endanger fiscal stability, and also welfare cost could be limited. When *r*<*g* governments can sustain higher levels of debt without need to raise taxes or cut spending, specifically, governments can run fiscal deficits and simultaneously face decreasing rate of public debt to gdp ratio (De Grauwe and Ji, 2019).

The public debt accumulation equation can be represented according to Blanchard (2019) as:


(7) $$\begin{array}{l} \frac{B_{t}}{Y_{t}}-\frac{B_{t-1}}{Y_{t-1}}=\left(r-g\right)\cdot \frac{B_{t-1}}{Y_{t-1}}+\frac{G_{t}-T_{t}}{Y_{t}} \end{array}$$

where:

- $\frac{B_{t}}{Y_{t}}$ is the public debt-to-GDP ratio at time *t*,
- *r* is the average interest rate on public debt,
- *g* is the nominal GDP growth rate,
- $\frac{G_{t}-T_{t}}{Y_{t}}$ is the primary deficit as a percentage of GDP.

Equation 7 indicates that there are two sources driving the dynamics of the public debt-to-GDP ratio: (i) condition of *r*<*g* (giving rise to a negative γ_1_), and (ii) given a primary deficit (or small or zero surplus): if *r*−*g*>0, public debt-to-gdp ratio will grow, exhibiting a positive γ_1_, unless the government primary surplus is high enough to counteract the debt growth. The results of the varying γ_1_ will be demonstrated in simulations, via employing NMPC and Deep Reinforcement Learning algorithm, described in the next section. It should be noted that differential Equations 5, 6 are reparametrized and modified versions of Equation 7, presented in structural form. Specifically, *r*−*g* is reparameterized as γ_1_. Additionally, a debt target is introduced, and instead of the primary deficit, the concept of fiscal surplus is used.

## 4 NMPC and reinforcement learning algorithms

Motivation for the section is to describe two algorithms, which we use to solve the macroeconomic model given in the previous section, these are: Non-linear Model Predictive Control and Deep Reinforcement Learning Algorithm, specifically, Soft Actor-Critic (SAC).

Historically, the algorithms we use originated from different research paradigms: the first from control theory, the latter from computation science. In his work Bertsekas (2024) identifies several similarities, between model predictive control and reinforcement learning algorithms, which is given below (Bertsekas, 2024). One similarity is that both MPC and some versions of RL are based on the principles of dynamic programming which is a mathematical optimization method used for solving complex problems by breaking them down into simpler subproblems. According to Bertsekas in MPC, dynamic programming principles are employed to solve a sequence of optimization problems over a finite horizon, where each solution provides an optimal control action based on the current state and predicted future states of the system. In principle, this is similar to the methods used in some versions of RL, where iterative techniques, such as value iteration and policy iteration, are employed to compute optimal actions that maximize expected cumulative reward over time (Bertsekas, 2022). Another similarity, is the use of sampling methods, for policy update and usage of iterative methods for policy update. In the context of MPC, policy is updated by solving optimization problem on the given horizon, via control action implementation. In the context of RL, policy update is implemented via sampling or simulation. Specifically, RL does so via generating possible future values of paths and maximizing expected future rewards considering different expected returns (Bertsekas, 2020).

### 4.1 NMPC

Non-linear Model Predictive Control (NMPC) is an optimization method developed for non-linear systems (Grüne and Pannek, 2017), and it is based on the respective solution of the control problems, given finite decision horizon (Johansen, 2011). Here we summarize, the idea of NMPC based on the works of Grüne and Pannek (2017) and Grüne et al. (2015). The discrete version of the problem can be described as follows: consider a system with its state represented by *x*_*n*_ at discrete time points *t*_*n*_. The aim is for *x*_*n*_ to follow an optimal reference trajectory, $x_{n}^{\text{ref}}$, as defined in Grüne and Pannek (2017). To achieve this, the state *x*_*n*_ is controlled using an input *u*_*n*_, which is given in feedback form, *u*_*n*_ = μ(*x*_*n*_). The function μ maps the state *x*∈*X* to the control set *U*.

Non-linear Model Predictive Control (NMPC) has the following form:


$$\begin{array}{l} x_{n+1}=f\left(x_{n},u_{n}\right) \end{array}$$

where *f*(*x*_*n*_, *u*_*n*_) is a non-linear function that governs how the system transitions from state *x*_*n*_ to the next state *x*_*n*+1_, based on the control input *u*_*n*_. This forms the basis for the control approach under consideration. The optimal control problem can be described as follows: the objective is to minimize a cost function *J*(*x*_0_, *u*(·)), where *x*_0_ represents the initial state. The cost function is expressed as the sum of stage costs over a time horizon of *N* steps, formulated as:


$$\begin{array}{l} J\left(x_{0},u\left(\cdot \right)\right):=\overset{N-1}{\underset{k=0}{\sum }}\ell \left(x_{k},u_{k}\right), \end{array}$$

where each stage cost ℓ(*x*_*k*_, *u*_*k*_) is defined as:


$$\begin{array}{l} \ell \left(x_{k},u_{k}\right)=||x_{k}|{|}^{2}+\lambda ||u_{k}|{|}^{2}. \end{array}$$

In this expression, the stage cost ℓ(*x*_*k*_, *u*_*k*_) penalizes deviations of the system state *x*_*k*_ from a reference trajectory, while also accounting for the control effort *u*_*k*_. The parameter λ weights the balance between minimizing the state error and limiting the control effort.

Furthermore, we can also formulate a related control problem involving the maximization of a discounted performance measure *J*_*N*_(*x*_0_, *u*), represented by:


$$\begin{array}{l} \underset{u\in U}{\max}J_{N}\left(x_{0},u\right)\;\text{where }J_{N}\left(x_{0},u\right):=\overset{N-1}{\underset{k=0}{\sum }}\beta^{k}g\left(x_{k},u_{k}\right). \end{array}$$

In this case, β^*k*^ is a discount factor applied at each time step *k*, and *g*(*x*_*k*_, *u*_*k*_) represents the stage performance criterion, which depends on both the system state *x*_*k*_ and the control input *u*_*k*_. The goal here is to maximize the cumulative discounted performance over the finite control horizon *N* (Grüne et al., 2015).

### 4.2 Basic description of reinforcement learning

Reinforcement learning is part of Artificial Intelligence, which is about “learning from interaction” (Sutton and Barto, 2018, p. 1). The Reinforcement Learning family of algorithms provide tools to for learning optimal policy in a sequential decision-making set-up. Optimality of the policy means choosing the policy that maximizes cumulative rewards. Before moving on describing the particular algorithm from the Deep Reinforcement Learning family, we give a simple graphical example, which describes the basic formulation of reinforcement learning and also provides a description of key concepts. For this, we use the graphical representation from Russell and Norvig (2010, p. 832) to Zhao (2024).

**Figure 6:**
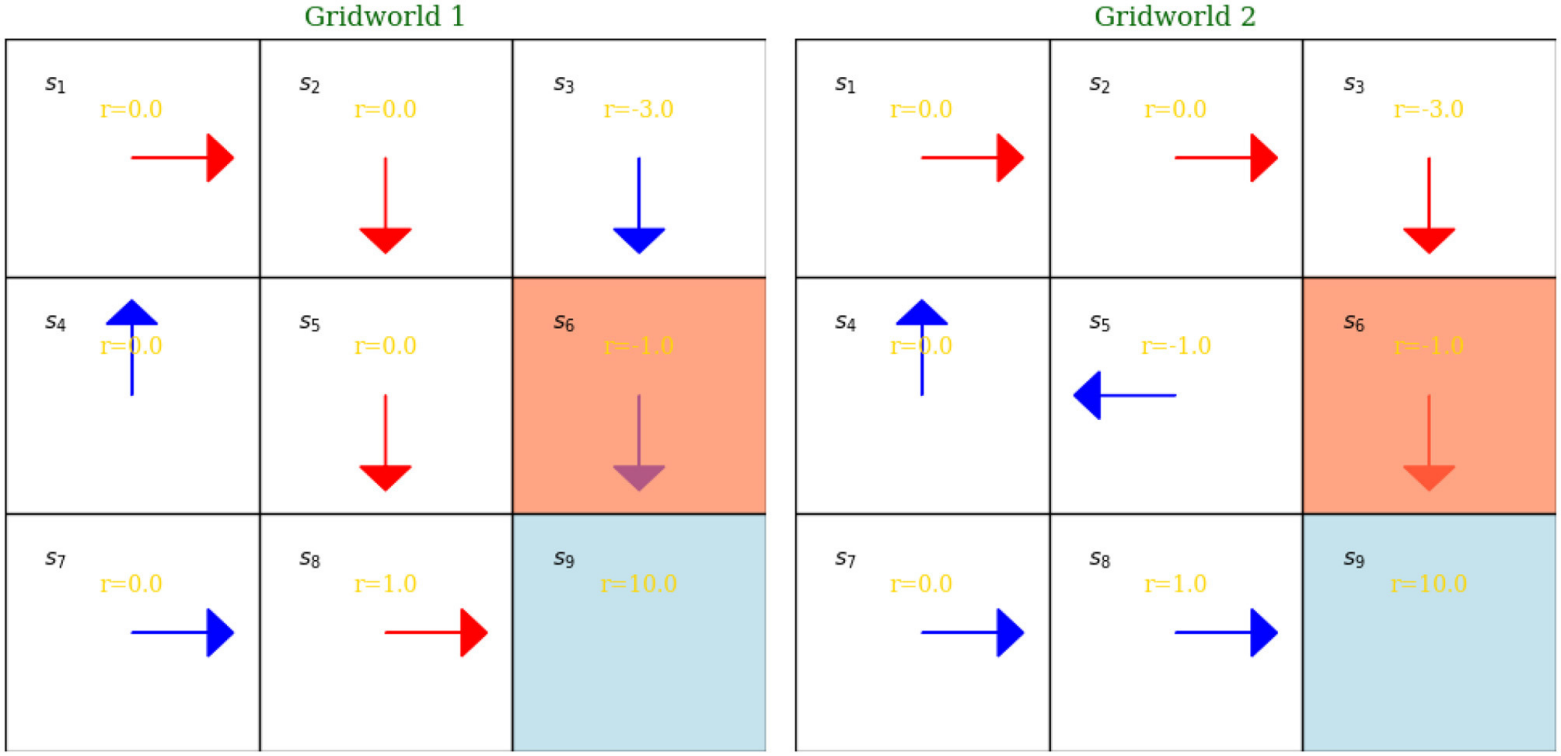
Two gridworld examples demonstrating RL concepts.

The plot in Figure 6 illustrates two simple gridworld examples, which serve as representations of the reinforcement learning (RL) concepts described in Box 1 above. Each gridworld consists of nine distinct states. Policies are illustrated using arrows. The gridworlds are populated with intermediate rewards, which can be either negative or positive. Both policies are deterministic, as the probability of taking a specific action given a state is one, as indicated by the arrow directions. The state *s*_9_ serves as the terminal state. For instance, the trajectory in the first gridworld, assuming the agent starts at *s*_1_, will be a collection of state-action-reward triplets leading to the terminal state *s*_9_. The policy represented by the red arrows in the first gridworld is superior to that of the second gridworld, as it yields a higher total reward.

Box 1 **Summary of key concepts in reinforcement learning.**

- **State space**: the set of all possible states, denoted as $S={\left\{s_{i}\right\}}_{i=1}^{n}$. Each state represents the current situation or condition of the environment.
- **Action space**: the set of all available actions, denoted as $A\left(s_{i}\right)={\left\{a_{i}\right\}}_{i=1}^{n}$. These are the actions the agent can choose from while in a given state.
- **Reward**: at each time step, the agent receives a reward, represented as a real number: $R_{t+1}\in R\subset \mathbb{R}$.
- **Policy**: the policy defines the probability of taking an action *a* given the agent is in state *s*. It is expressed as a conditional probability: π(*a*∣*s*). Policies can be either deterministic or stochastic. A stochastic policy means there may be different probabilities for taking various actions in a given state.
- **Trajectory**: a sequence of state, action, and reward triplets that describe the agent's path through the environment.
- **Agent**: the decision-maker that selects actions based on its policy.
- **Environment**: everything outside the agent can be considered as environment. The agent interacts with the environment by performing actions, which may or may not result in transitioning to a different state. By acting, the agent may transition to a new state and receive feedback in the form of scalar-valued rewards.
- **Goal**: the agent's goal is to maximize the total cumulative reward. If discounting is applied, the goal is to maximize the expected discounted return. This is controlled by the discount factor γ (in the macroeconomic context given in previous section, γ_1_ is used to represent different coefficient). The discounted return is defined as:
  $$G_{t}≐R_{t+1}+\gamma R_{t+2}+\gamma^{2}R_{t+3}+\cdots =\overset{\infty }{\underset{k=0}{\sum }}\gamma^{k}R_{t+k+1},$$
- where *G*_*t*_ is the expected return, and 0 ≤ γ ≤ 1 is the discount rate, which regulates rewards got earlier is more valuable than rewards obtained later (Sutton and Barto, 2018). This parameter is different from "algorithmic discount factor" λ as discussed in Powell (2022, p. 51).

In the taxonomy of the Reinforcement Learning from the perspective of policy optimization there are two major approaches which are widely adopted: (i) value-based methods and (ii) policy-based methods. These approaches use function approximations, unlike the classical reinforcement learning models which are based on a tabular approach. Function approximations are important given inefficiencies related to tabular representations under complex action space (specifically, in terms of memory requirements and computational complexity) (Ding et al., 2020). Value-based methods are centered around the concept of learning value functions, which estimate the cumulative reward that an agent can achieve starting from a given state and taking a specific action in a state. These methods seek to determine the optimal policy indirectly by first finding the optimal value function. Most prominent algorithms from the family of value-based methods are Q-learning and Deep Q-Network (DQN). The two primary types of value functions used in this approach are the state value function, *V*_π_(*s*), and the action value function, *Q*_π_(*s, a*). The state value function estimates the expected return starting from state *s* and following a particular policy π. Formally, it is defined as:


(8) $$\begin{array}{l} V_{\pi }\left(s\right)=E_{\pi }\left[\overset{\infty }{\underset{t=0}{\sum }}\gamma^{t}r_{t+1}|s_{t}=s\right], \end{array}$$

where γ∈[0, 1) is the discount factor that establishes the present value of future rewards, and *r*_*t*+1_ is the reward received at time step *t*+1. The action value function, *Q*_π_(*s, a*), estimates the expected return for taking an action *a* in state *s* and subsequently following policy π. It is defined as:


(9) $$\begin{array}{l} Q_{\pi }\left(s,a\right)=E_{\pi }\left[\overset{\infty }{\underset{t=0}{\sum }}\gamma^{t}r_{t+1}|s_{t}=s,a_{t}=a\right]. \end{array}$$

Value-based methods often employ the Bellman equation to recursively compute these value functions. The Bellman equation defines the connection between the value of a state and the values of its successor states, which is fundamental to algorithms such as Dynamic Programming, and Temporal Difference learning. On the other hand DQN approximates action-value functions with the use of neural networks (Sutton and Barto, 2018). Value-based methods are useful given they have certain advantages, such as sample efficiency and low variance. On the other hand one of the disadvantage of the value-based methods are that they are not designed for problems which require a continuous action space (Zhang and Yu, 2020), and in macroeconomics this sort of problems are common.

In contrast, policy-based methods directly parameterize the policy and optimize it to maximize the expected cumulative reward. In other words, the policy update process stops only when the cumulative reward is maximized (Zhang and Yu, 2020). These methods do not require an explicit representation of the value function; instead, they focus on finding an optimal policy by directly optimizing the parameters that define the agent's behavior (Sutton and Barto, 2018). A policy function, denoted as π_θ_(*a*∣*s*), defines the probability of taking action *a* given state *s* under parameter θ. Policy-based methods optimize this function to maximize the expected reward, which can be achieved using gradient ascent algorithm. Deep Reinforcement Learning represents the integration of Reinforcement Learning and function approximator, which is frequently neural networks (Haarnoja et al., 2018b). Most prominent ones from the family of policy-based algorithms are TRPO (Trust Region Policy Optimization), PPO (Proximal Policy Optimization) and PG (policy gradient methods). Unlike value-based methods, policy-based methods work well for the problems, which have continuous action space and also have advantage in convergence (Zhang and Yu, 2020).

### 4.3 Soft Actor-Critic (SAC)

Combination of the two types of algorithms in terms of policy optimization described in the previous section is called actor-critical algorithms. In this family of algorithms, the actor is equivalent to a policy under which decisions are being made. On the other hand, critic is the value function which assesses actions produced by chosen policy produced by an actor. Usually, policy and value functions are neural networks (Powell, 2022). Actor-critic algorithms utilize value-based approach to learn the Q-function, and the policy-based approach to learn policy function. By doing so, actor-critic algorithms utilizes advantages of both valued-based approach in terms of increasing sample efficiency and policy-based methods for making the algorithm applicable to a continuous action space (Zhang and Yu, 2020). One of the algorithms from the actor-critic family of algorithms is Soft Actor-Critic (SAC). Soft Actor-Critic (SAC) was first introduced by Haarnoja et al. (2017) as an energy-based reinforcement learning algorithm, and was further developed in Haarnoja et al. (2018a) and Haarnoja et al. (2018b). According to the authors, SAC addresses two major issues that previous algorithms struggled with: sample efficiency and the complexity of choosing the most effective hyperparameters. One of the characteristics of the SAC is that it has two objectives: maximization of both returns and entropy at the same time. As the authors of the algorithm put it, the goal is to complete tasks as successfully as possible and as randomly as possible Haarnoja et al. (2018b, p. 1). In SAC, “soft” refers to entropy regularization, which is introduced to encourage exploration. A higher level of entropy implies a broader range of action choices. The entropy term helps prevent the stochastic policy from collapsing into a deterministic one, which is important for avoiding convergence to suboptimal local optima. If entropy regularization is removed, SAC effectively reduces to the performance of the TD3 algorithm, while the mechanics of the two algorithms are still different (Sigaud, 2020).

In the context of Deep RL, sample efficiency refers to the requirement that on-policy algorithms, such as PPO or its predecessor (TRPO), need new samples for each update. On-policy methods learn the best value of a policy from the given policy, and the update entails interactions of the same policy. In other words, PPO collects trajectories under current policy and updates policy on the given data. In terms of sample efficiency, it means that collected data points are used only once for the training. For the case of Off-Policy algorithms (and there two oft-used: DQN and SAC), the algorithms learn from experiences collected under different policies. SAC uses a replay buffer, where it stores past interactions for training process, which makes it more sample efficient (Zhang et al., 2020).

SAC is based on the maximum entropy regularization method^4^, and here we provide a brief overview based on the paper (Haarnoja et al., 2018b). The goal of the algorithm is to learn a policy π(***a***_*t*_∣***s***_*t*_) that maximizes the expected reward. The augmented objective function, which includes entropy, has the following form:


(10) $$\begin{array}{l} \pi^{*}=\arg\underset{\pi }{\max}\underset{t}{\sum }E_{\left(s_{t},a_{t}\right)\sim \rho_{\pi }}\left[r\left(s_{t},a_{t}\right)+\alpha H\left(\pi \left(\cdot |s_{t}\right)\right)\right] \end{array}$$

The augmented objective function aims to maximize both the expected return and entropy. α denotes the temperature parameter, which acts as a weight for entropy (indicating how much importance is given to entropy compared to the return). Therefore, if α approaches zero, the entropy-augmented objective function reduces to the standard RL objective function. For the optimization, SAC uses two networks: (i) soft Q-function, and (ii) Policy Network. Q-function network *Q*_θ_(***s***_*t*_, ***a***_*t*_) estimates the expected return by taking action ***a***_*t*_ in a state ***s***_*t*_ under the current policy π_ϕ_. Policy (actor) network π_ϕ_(***a***_*t*_∣***s***_*t*_) is parameterized by ϕ, and this network represents the policy, mapping states ***s***_*t*_ to a distribution over actions ***a***_*t*_. The policy is parameterized as a Gaussian distribution whose mean and covariance are produced by a neural network (Zhang et al., 2020). Minimization of the following cost function, also known as the soft Bellman residual, provides the parameters for the soft Q-function:


(11) $$\begin{array}{l} J_{Q}\left(\theta \right)=E_{\left(s_{t},a_{t}\right)\sim D}\left[\frac{1}{2}{\left(Q_{\theta }\left(s_{t},a_{t}\right)-\hat{Q}\left(s_{t},a_{t}\right)\right)}^{2}\right], \end{array}$$

where:

- *Q*_θ_(***s***_*t*_, ***a***_*t*_) is the current estimate of the Q-function, parameterized by θ.
- $\hat{Q}\left(s_{t},a_{t}\right)$ is the **soft Bellman backup target**.

The cost function (Equation 11) consists of two components: the **current Q-function estimate** and the soft Bellman backup target, which is defined as:


$$\begin{array}{l} \hat{Q}\left(s_{t},a_{t}\right)=r\left(s_{t},a_{t}\right)+\gamma E_{s_{t+1}\sim p}\left[V_{\bar{\theta }}\left(s_{t+1}\right)\right]. \end{array}$$

By substituting the soft value function $V_{\bar{\theta }}$, the cost function can be rewritten as:


(12) $$\begin{array}{l} J_{Q}(\theta )=E_{\left(s_{t},a_{t}\right)\sim D}[\frac{1}{2}(Q_{\theta }\left(s_{t},a_{t}\right)-(r\left(s_{t},a_{t}\right)\\ \;{+\gamma E_{a_{t+1}\sim \pi }\left[Q_{\bar{\theta }}\left(s_{t+1},a_{t+1}\right)-\alpha \log\pi \left(a_{t+1}|s_{t+1}\right)\right]))}^{2}] \end{array}$$

The value function is parameterized by the soft Q-function and is expressed as:


$$\begin{array}{l} V_{\bar{\theta }}\left(s_{t}\right)=E_{a_{t}\sim \pi_{\phi }}\left[Q_{\bar{\theta }}\left(s_{t},a_{t}\right)-\alpha \log\pi_{\phi }\left(a_{t}|s_{t}\right)\right] \end{array}$$

where α controls the trade-off between reward maximization and entropy (exploration).

The cost function (Equation 11) is optimized using **stochastic gradient descent**. The gradient of the cost function is:


(13) $$\begin{array}{l} {\hat{\nabla }}_{\theta }J_{Q}(\theta )=\nabla_{\theta }Q_{\theta }\left(s_{t},a_{t}\right)(Q_{\theta }\left(s_{t},a_{t}\right)-(r\left(s_{t},a_{t}\right)\\ \;+\gamma \left(Q_{\bar{\theta }}\left(s_{t+1},a_{t+1}\right)-\alpha \log\pi_{\phi }\left(a_{t+1}|s_{t+1}\right)\right))). \end{array}$$

Policy network parameters are optimized by minimizing the following cost function:


$$\begin{array}{l} J_{\pi }\left(\phi \right)=E_{s_{t}\sim D}\left[{\text{D}}_{\text{KL}}\left(\pi_{\phi }\left(\cdot |s_{t}\right)||\frac{\exp\left(Q_{\theta }\left(s_{t},\cdot \right)\right)}{Z_{\theta }\left(s_{t}\right)}\right)\right], \end{array}$$

where D_KL_ represents the Kullback-Leibler (KL) divergence, a measure of the difference between two probability distributions. This procedure aligns the policy π_ϕ_ with the target distribution defined by the soft Q-function, $\frac{\exp\left(Q_{\theta }\right)}{Z_{\theta }}$, where *Z*_θ_ is the partition function. While *Z*_θ_ normalizes the distribution, it does not depend on ϕ and can be ignored during optimization.

The objective can be rewritten in the following form:


(14) $$\begin{array}{l} J_{\pi }\left(\phi \right)=E_{s_{t}\sim D}\left[E_{a_{t}\sim \pi_{\phi }}\left[\alpha \log\left(\pi_{\phi }\left(a_{t}|s_{t}\right)\right)-Q_{\theta }\left(s_{t},a_{t}\right)\right]\right]. \end{array}$$

Here, the term αlogπ_ϕ_(***a***_*t*_∣***s***_*t*_) encourages exploration by maximizing entropy, while the term −*Q*_θ_(***s***_*t*_, ***a***_*t*_) encourages the policy to choose actions with higher expected returns. The parameter α is the entropy scaling factor that balances exploration and exploitation.

To optimize this objective, the SAC algorithm computes its gradient using the reparameterization trick. It is important to note that in Equation 14, the expectation is taken over actions. The reparameterization trick allows to express the sampled actions ***a***_*t*_ as a deterministic function of noise ϵ_*t*_ drawn from a standard Gaussian distribution. Because the expectation is now taken with respect to a fixed noise distribution independent of the parameters ϕ, it allows backpropagation through the sampling process. Specifically, the action is computed as


$$a=\tanh\left(\mu_{\phi }+\epsilon \cdot \sigma_{\phi }\right),\;\text{where }\epsilon \sim N\left(0,1\right),$$

and the expectation is reformulated over ϵ instead of *a* (Sigaud, 2020). In the context of continuous action spaces, this reparameterization provides a lower-variance gradient estimator for training the policy network (Zhang et al., 2020).

The final gradient of the objective is:


(15) $$\begin{array}{l} {\hat{\nabla }}_{\phi }J_{\pi }(\phi )=\nabla_{\phi }\alpha \log\left(\pi_{\phi }\left(a_{t}|s_{t}\right)\right)+(\nabla_{a_{t}}\alpha \log\left(\pi_{\phi }\left(a_{t}|s_{t}\right)\right)\\ \;-\nabla_{a_{t}}Q_{\theta }\left(s_{t},a_{t}\right))\nabla_{\phi }f_{\phi }\left(\epsilon_{t};s_{t}\right). \end{array}$$

Here, ***a***_*t*_ = *f*_ϕ_(ϵ_*t*_; ***s***_*t*_) is the reparameterized action, where *f*_ϕ_ maps the input noise ϵ_*t*_ to the action space based on the policy network's parameters. The term ϵ_*t*_ is a noise vector sampled from a Gaussian distribution N(0,I). The first term, ∇_ϕ_αlog(π_ϕ_(***a***_*t*_∣***s***_*t*_)), accounts for the direct dependency of the policy on ϕ. The second term includes gradients with respect to actions ***a***_*t*_, backpropagated through the policy network. This formulation ensures that the gradient computation respects both the stochastic nature of the policy and the dependencies introduced by the Q-function. As mentioned at the beginning of this section, the SAC algorithm is appealing due to its ability to automatically tune hyperparameters. Specifically, the entropy coefficient α, which regulates the exploration-exploitation trade off, is adjusted dynamically. To explain the process of automatic tuning, we adopt a simplified and intuitive approach, as presented in Morales (2020).

The objective function for α is given as:


(16) $$\begin{array}{ll} J\left(\alpha \right) & =E_{s\sim U\left(D\right),â\sim \pi }\left[\alpha \left(H+\log\pi \left(â|s;\phi \right)\right)\right]. \end{array}$$

In this process, states are sampled from the replay buffer, and actions are drawn from the policy. The negative of this objective is minimized to maximize the weighted sum of the target entropy H and the log probability of the policy, scaled by α. This dynamic adjustment ensures an optimal balance between exploration and exploitation (Zhang et al., 2020).

## 5 Comparative analysis of NMPC and SAC simulation results

In this section we provide a comparative analysis of the NMPC and SAC simulations results. Firstly, we analyze baseline simulation results, and next we show different scenarios for checking robustness in terms of initial values and varying parameters. We present both state and control variable in the following graphs.

### 5.1 SAC simulation results

**Figure 7:**
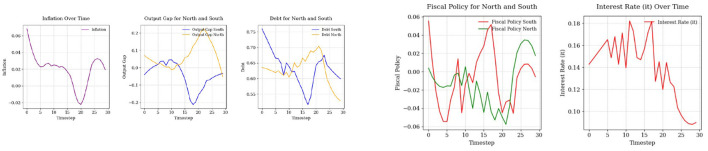
Comparison of state variables **(left)** and control variables **(right)** over time.

For the simulation process, we use the environment built on the equations described in the third section of the paper. To implement the SAC algorithm, we used a custom SAC agent developed in Python and, separately, the stable-baselines3 implementation (Raffin et al., 2021). As shown in Figure 7, inflation demonstrates a controlled trajectory that remains within relatively stable bounds. Starting with an initial value 0.06, inflation initially exceeds the target of π^*^ = 0.02, however, the SAC algorithm effectively guides policy adjustments, causing inflation to steadily decline and move closer to the target. During the middle phase (time steps 6–20), inflation dips below the target, reaching slightly negative values, which may indicate a temporary deflationary pressure. In the final phase (time steps 21–30), inflation gradually rises and moves toward the target of 0.02. The inflation trajectory highlights the SAC algorithm's effectiveness in achieving price stability. While inflation starts at a higher level, it is progressively brought closer to the target, showcasing the algorithm's capacity to regulate inflation within normal bounds over time.

The output gaps for both the North and South regions eventually stabilize near the target of zero, demonstrating satisfactory policy outcomes. However, the South's greater variability underscores the need for enhanced measures to improve economic stability and resilience to shocks. In the North, the output gap begins positively at around 0.1, indicating a slight economic overheating relative to the potential output. This quickly stabilizes, hovering near zero by time step 5. In the final phase (time steps 21–30), the output gap peaks briefly at 0.25 around time step 22, before returning back closer to zero by time step 30. Overall, the North's output gap is well-managed throughout the period, with deviations from zero being relatively small and short-lived. In contrast, the South's output gap starts close to zero but exhibits slightly larger fluctuations than the North during the initial phase (time steps 0–5), reaching a minimum of ~ −0.1 and peaking briefly around 0.05. During the middle phase (time steps 6–20), the South shows higher volatility compared to the North. In the final phase (time steps 21–30), the South's output gap experiences notable swings, peaking at around −0.2 before ultimately trending toward zero. Despite this volatility, the region gradually aligns with the target by the end of the period, demonstrating the effectiveness of policy adjustments over time.

The debt trajectories for both the North and South regions start above the target of d^*^ = 0.6. The South region's debt level is higher, reflecting the economic realities of Southern EU countries in that initial period, which often experienced greater fiscal pressures compared to their Northern counterparts. While the South's debt peaks above 0.75 at the beginning, yet, the debt in the South adjusts, while the North's debt starts slightly above 0.65 and then moves down.

Throughout the observed period, the SAC algorithm successfully manages to reduce debt levels in both regions. For the South, the debt trajectory exhibits volatility, initially declining sharply, then oscillating before gradually moving toward the target. In the final phase, the South's debt moves closer to d^*^ = 0.6, although it remains slightly above the target. In the North, the debt trajectory follows a smoother downward trend. After a brief initial decline, the debt level stabilizes and converges below the target.

Both control variables are used actively to move the state variables closer to their targets. Until time step 17, monetary policy acts aggressively, possibly to bring the inflation rate closer to its target, before it begins to decline. The movement of fiscal balance plays an important role in controlling both output gaps and debt dynamics. In the final steps, fiscal policy for both the North and South adheres to the EU fiscal policy rules, but with some fiscal consolidation policies. For the South, fiscal policy is slightly negative, while for the North, it stabilizes around 2%. This alignment demonstrates the effectiveness of the SAC algorithm in maintaining compliance with fiscal rules while targeting economic stability.

**Figure 8:**
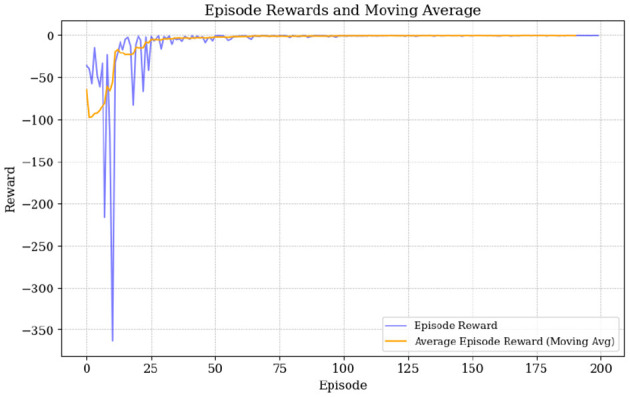
Episode rewards and moving average.

Figure 8 demonstrates how the SAC algorithm effectively stabilizes rewards over time, which shows successful policy optimization. Until the 50th episode, cumulative rewards experience significant fluctuations, which is expected given the complexity of the macroeconomic environment and the existence of a continuous action space. However, after 70 episodes, the rewards stabilize closer to the target (minimizing negative rewards), indicating a successful outcome and highlighting the effectiveness of the SAC algorithm in achieving both good exploration and debt stabilization results (although with some fiscal consolidation cost). Given this observation, the number of episodes was restricted to 200, as in several simulations, an excessively high number of episodes led to instability in the algorithm's performance. Also note that the parameter γ_1_ is kept negative for baseline model (assuming that *r*<*g*) which will be change below, see section 6.

### 5.2 NMPC simulation results

For the simulation of the control problem, we use *do-mpc*, a tool in Python developed by Fiedler et al. (2023). *do-mpc* is a tool for linear, non-linear, and robust model predictive control. The results under the NMPC algorithm demonstrates smoother and well-controlled trajectories, reflecting its deterministic nature and ability to optimize policy decisions without the randomness inherent in stochastic methods like SAC. Below is a detailed analysis of both the control and state variable trajectories.

**Figure 9:**
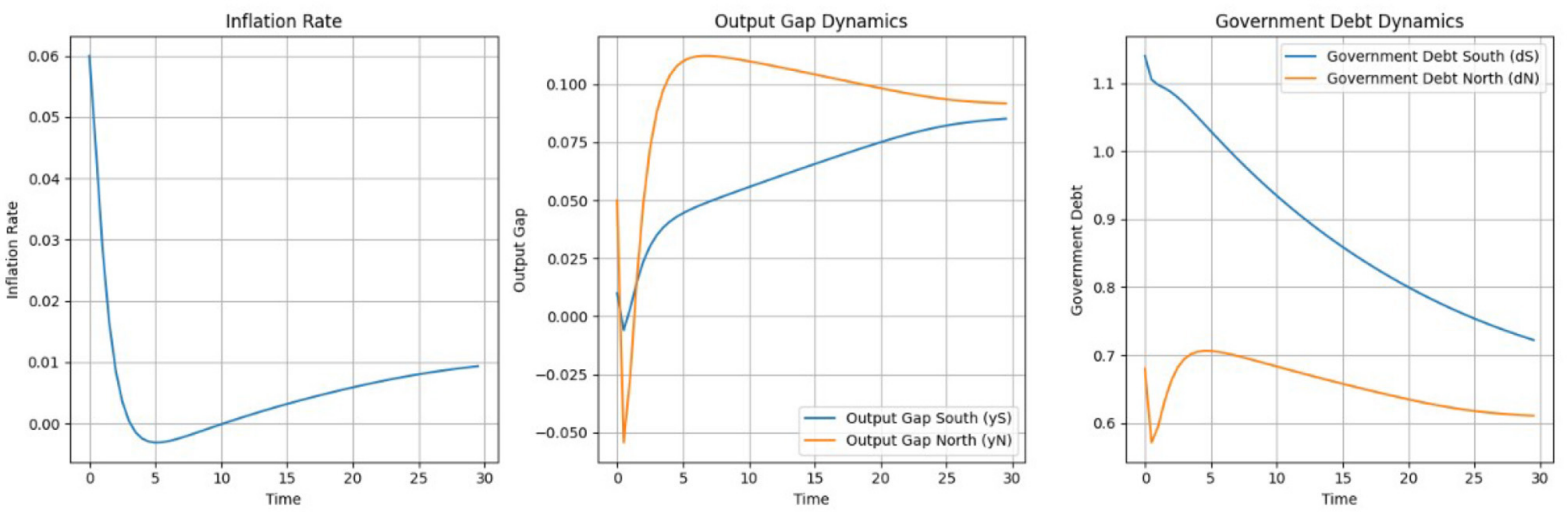
State variables under NMPC: inflation, output gap, and government debt dynamics.

Assuming again that *r*<*g* and thus a positive γ_1_as shown in Figure 9, the NMPC algorithm achieves stable adjustments for the interest rate and fiscal policy. The interest rate starts at zero and remains stable for the first 15 time steps. After time step 15, the interest rate begins to rise steadily, reaching ~ 0.014 by time step 30. This controlled increase highlights NMPC's capacity to manage monetary policy effectively without introducing abrupt changes. Fiscal policy adjustments are also smooth, with both the North and South regions converging to steady levels by the end of the simulation. Fiscal policy for the North stabilizes slightly above zero, around 0.02, reflecting a slight surplus. For the South, fiscal policy remains marginally negative, around −0.02, meaning fiscal consolidation, and likely supporting debt stabilization and output gap reduction. As in the case of the SAC these adjustments are in line with some the EU actual fiscal policies.

**Figure 10:**
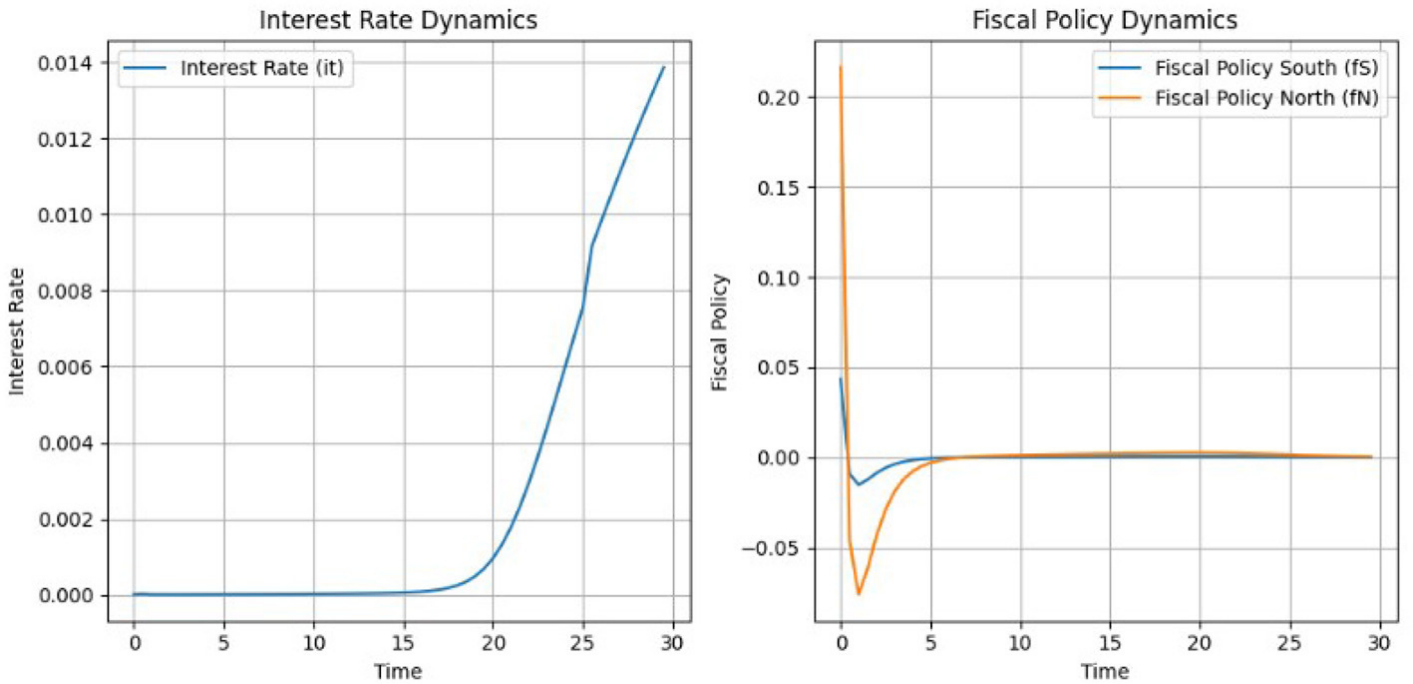
Interest rate and fiscal policy dynamics under NMPC.

Figure 10 demonstrates the trajectories of key state variables—inflation, output gaps, and government debt—under NMPC. We used the same initial values for the state variables under both SAC and NMPC. The inflation rate starts at ~ 0.06, well above the target of 0.02. The algorithm reduces inflation in the early phase, reaching a minimum close to zero by time step 10. Afterward, inflation gradually converges toward the target, reaching ~0.02 by time step 30. As with the SAC algorithm, the output gap remains at a relatively less controllable state variable under NMPC. The output gap for the North stabilizes at ~0.87, while for the South, it stabilizes at around 0.8. This indicates that while NMPC provides smoother trajectories, achieving precise control over the output gap remains a challenge.

Government debt levels show consistent and steady declines in both regions. The South starts with a higher debt-to-GDP ratio, exceeding 1.1. Debt declines smoothly, converging closer to 0.7 by the end of the simulation. The North, with an initial debt level of 0.7, achieves faster stabilization and converges toward the target of 0.6. Across multiple simulations and under varying weights, the results demonstrate that SAC is more effective at reducing debt below the target level in both the South and North regions under the stable (baseline) scenario. Specifically, as shown in Figure 7, the debt indicator for the South reaches the target level, while the indicator for the North falls below it. In contrast, for the NMPC version shown in Figure 10, while the algorithm successfully brings the Norths debt indicator close to the target, it struggles to achieve the same for the South.

The NMPC framework excels in generating smooth and predictable trajectories for all variables. The interest rate and fiscal policies for both regions are adjusted with minimal volatility, ensuring compliance with macroeconomic objectives and EU fiscal policy rules. Inflation, output gaps, and debt trajectories converge steadily toward their respective targets, reflecting effective policy management. Overall, the deterministic nature of NMPC ensures precise and stable economic adjustments, making it a robust tool for macroeconomic stabilization compared to stochastic alternatives like SAC.

While NMPC provides more stable and smooth trajectories, the inherent fluctuations in the SAC results may offer a more realistic depiction of how economic variables evolve in the face of ongoing policy adjustments, market reactions, and internal and external shocks. The fluctuations seen in the SAC results may illustrate how the algorithm navigates these trade-offs, adjusting policies dynamically in response to changing priorities, conditions and shocks. Economic policies also often have delayed effects (Chen et al., 2022). The fluctuations in the SAC outcomes could reflect these lags in policy impact, where initial policy actions cause immediate fluctuations, followed by further adjustments as the delayed effects manifest. The SAC results demonstrate more short-term volatility, which can reflect real-economies situations where economic indicators such as inflation and output gaps do not adjust smoothly but instead fluctuate due to immediate market reactions, consumer sentiment, investor behavior and external shocks.

The SAC algorithm relies on reinforcement learning, which involves with continuous adaptation and learning from interactions with the environment. This method can inherently capture the trial-and-error nature of economic policy making, where decisions are made based on current conditions and then adjusted as new information becomes available. The resulting fluctuations can mirror the constant adjustments policymakers make in response to economic indicators. However, NMPC requires a comprehensive and accurate model of the economic system's dynamics, which can be a limitation when dealing with highly complex or evolving environments. The quality of the control actions generated by NMPC is directly tied to the accuracy of the underlying model. In cases where the model is incomplete or fails to capture certain non-linearities or stochastic elements, NMPC's performance may degrade.

## 6 Debt dynamics under SAC

In this section, we present the debt dynamics under different scenarios of *r*−*g*, specifically focusing on the varying signs of γ_1_, which regulate the debt dynamics for the North and South regions, in the face of different debt equilibria. From Blanchard's debt equation discussed earlier, debt sustainability is achieved, facing a good debt equilibrium, when the interest rate is lower than the growth rate, and the primary balance is zero or close to zero.

**Figure 11:**
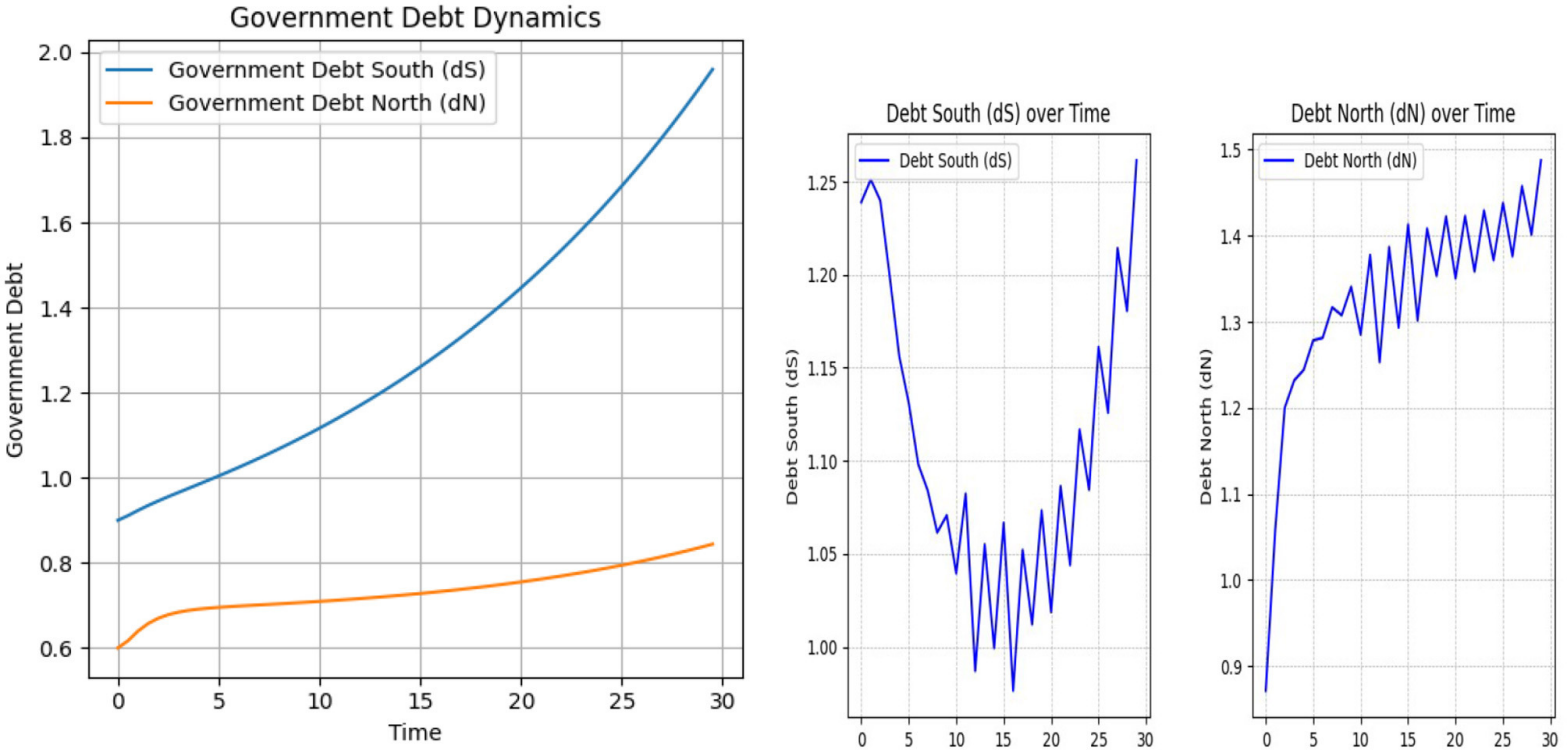
Debt South (dS) and Debt North (dN) dynamics under positive γ_1_ settings: on the left, NMPC results; on the right, SAC results.

In the context of the differential Equations 5, 6, we assume that the γ_1_ coefficient will be positive, i.e., when *r*>*g*, leading to debt instability. This instability is usually pursued and mitigated through austerity policies, meaning $f_{t}^{S}$ and $f_{t}^{N}$ must be positive. Here, we demonstrate this assumption using the SAC and NMPC algorithms for the macroeconomic environment described in previous sections. The Figure 11 shows the case where γ_1_ is identical for both the South and North regions and is positive.

As shown in Figure 11, the debt dynamics differ significantly depending on whether the γ_1_ coefficient is negative or positive. On the left, we observe the NMPC results, which demonstrate that under a positive γ_1_, the algorithm is no longer able to stabilize the debt level close to the target. Moreover, for the South, the debt-to-GDP ratio follows an explosive trajectory. On the right, the SAC algorithm results are shown under the same parameter setting. Specifically, after an initial drop, the debt of the South surges to 125 percent by the end of 30 steps, while in the North, debt increases to over 150 percent by the end of the episode. These patterns illustrate how the sign of γ_1_ contributes to stability under Deep RL simulations and further support assumptions in macroeconomic literature regarding debt dynamics.

## 7 Conclusion

With this paper, we contribute to the literature on sovereign debt control in Europe, simulating debt dynamics in Eurozone countries and the application of deep reinforcement learning in macroeconomics. To our knowledge, this paper is the first attempt to apply machine learning in the form of the Soft Actor-Critic (SAC) algorithm in the context of a macroeconomic policy problem. Specifically, we present a novel approach to solving a multi-objective macroeconomic problem aimed at minimizing the deviations of multiple macroeconomic state variables from their target levels. Our approach utilizes Deep Reinforcement Learning alongside an established Non-linear Model Predictive Control (NMPC) framework from the macroeconomic literature to minimize deviations in the inflation rate, output gap, and debt levels under a cooperative scenario.

We further demonstrate that, while NMPC provides more stable and smooth trajectories, the inherent fluctuations observed in SAC results may offer a more realistic depiction of how economic variables evolve in response to ongoing policy adjustments, market reactions, and internal and external shocks and disruptions. These fluctuations illustrate how the SAC algorithm dynamically adjusts policies to navigate trade-offs, responding to changing priorities, conditions and shocks. Economic policies often exhibit delayed effects, and the SAC results demonstrate short-term volatility, reflecting real-economies situations where economic indicators such as inflation and output gaps do not adjust smoothly but instead fluctuate due to immediate market reactions, consumer sentiment, investor behavior and shocks and disruptions through local or global news (or misinformation).

The SAC algorithm, which belongs to deep reinforcement learning algorithm family, involves continuous adaptation and learning from interactions with the environment. This method inherently captures the trial-and-error nature of economic policy making, where decisions are made based on current conditions and subsequently adjusted as new information becomes available. The resulting fluctuations could mirror the constant adjustments policymakers make in response to evolving economic indicators and information.

Additionally, we explore how changes in parameters regulating debt dynamics—specifically those related to the difference between the interest rate and growth rate—can cause instability in debt trajectories. Our results highlight how the sign of γ_1_ influences stability in Deep RL simulations and further support macroeconomic literature assumptions regarding debt dynamics. Finally, we emphasize the importance of utilizing modern technical tools, including simulations powered by machine learning, to address macroeconomic management challenges. We hope this paper contributes meaningfully to the ongoing academic discussion in this direction.

## Data Availability

The original contributions presented in the study are included in the article/supplementary material, further inquiries can be directed to the corresponding authors.

## Footnotes

^1^For the long delay effects of interest rate changes, see Chen et al. (2022).

^2^See Grauwe (2025).

^3^The headline deficit, which includes interest payments, is the EU's main fiscal benchmark under the 3% of GDP rule in the SGP. It often exceeds the primary deficit, which excludes interest payments, especially when interest costs are high.

^4^Entropy regulates randomness in this context: higher entropy means higher randomness. For instance, if a coin is fair, the randomness and, consequently, the entropy will be higher.
